# Macrophage-compatible magnetic achiral nanorobots fabricated by electron beam lithography

**DOI:** 10.1038/s41598-022-17053-x

**Published:** 2022-07-29

**Authors:** Teng Jiang, Xiaoxia Song, Xueliang Mu, U. Kei Cheang

**Affiliations:** 1 Department of Mechanical and Energy Engineering, Southern University of Science and Technology, Shenzhen, 518055 China; 2 Shenzhen Key Laboratory of Biomimetic Robotics and Intelligent Systems, Southern University of Science and Technology, Shenzhen, China; 3 Guangdong Provincial Key Laboratory of Human-Augmentation and Rehabilitation Robotics in Universities, Southern University of Science and Technology, Shenzhen, China; 4 Department of Mechanical and Manufacturing Engineering, University of Calgary, Calgary, AB Canada

**Keywords:** Biomedical engineering, Mechanical engineering, Magnetic properties and materials

## Abstract

With the development and progress of nanotechnology, the prospect of using nanorobots to achieve targeted drug delivery is becoming possible. Although nanorobots can potentially improve nano-drug delivery systems, there remains a significant challenge to fabricating magnetically controllable nanorobots with a size suitable for drug delivery in complex in vivo environments. Most of the current research focused on the preparation and functionalization of microscale and milliscale robots due to the relative difficulties in fabricating nanoscale robots. To address this problem and move towards in vivo applications, this study uses electron beam lithography to fabricate achiral planar L-shaped nanorobots that are biocompatible with immune cells. Their minimal planar geometry enabled nanolithography to fabricate nanorobots with a minimum feature size down to 400 nm. Using an integrated imaging and control system, the locomotive behavior of the L-shaped nanorobots in a fluidic environment was studied by examining their velocity profiles and trajectories. Furthermore, the nanorobots exhibit excellent cell compatibility with various types of cells, including macrophage cells. Finally, the long-term cell culture medium immersion test demonstrated that the L-shaped nanorobots have robust stability. This work will demonstrate the potential to use these nanorobots to operate in vivo without triggering immune cell responses.

## Introduction

Wireless micro/nanobots have great potential to be used in applications in the biomedical and bioengineering fields, such as cargo delivery, targeted therapy, minimally invasive surgery^1,2^, and remote sensing^3–5^, because their small sizes allow them to navigate deep inside hard-to-reach areas of the body^6,7^. In recent years, a large number of mobile micro/nanorobots have been developed with different control methods^8^, including magnetic^9,10^, electric^11^, acoustic^12^, thermal^13^, and chemical^14^. The potential to use magnetic microrobots that can generate forward thrust when actuated using a rotating magnetic field (RMF) has been well reported in recent years. In particular, magnetic microrobots have been among the most studied due to the ease of using magnetic fields for remote control at low Reynolds number^15^. However, there are only a few works in recent years that addressed the critical parameters for in vivo applications, including size, biocompatibility, mobility, and targeting capability of the micro/nanorobots.

Among the significant criteria for designing medical micro/nanorobots for in vivo drug delivery, the size requirement for certain has been difficult to address due to limitations in nanofabrication^16,17^. Micro/nanorobots must be small enough to navigate pathways or spaces inside the body in order to reach the diseased region^18^. Furthermore, micro/nanorobots should be mass-manufacturable at a low cost. Numerous methods were proposed to fabricate magnetic micro/nanorobots; most notably, the direct laser writing (DLW) method enabled by the two-photon polymerization (2PP) technology allowed for the fabrication of almost any desirable shapes with a wide range of material and surface properties. DLW can create microrobots down to 100 nm in length with geometries favorable for low Reynolds number propulsion and can be functionalized for in vivo applications^19^; however, their micron size ultimately limited their synergy with nanomedicine. The use of DLW was necessary to create microrobots with 3D geometrical designs, such as helices, which limited the types of fabrication techniques that can be used since many nanofabrication techniques cannot easily fabricate complex 3D nanostructures. While glancing angle deposition (GLAD) can overcome these limitations, this method had only demonstrated the fabrication of helical micro/nanorobots. Achiral microrobots were introduced as an alternative design to minimize the geometrical requirements for low Reynolds number propulsion in hopes of lowering the requirements for fabrication^20^. Previous reports showed that achiral microrobots can swim at low Reynolds number under magnetic actuation and can potentially compete with helical structures speed-wise^21^. Photolithography was later used to create achiral planar microrobots with mass-manufacturability at low cost for drug delivery; however, this method was restricted to microscale geometrical features^22,23^. Building on the concept of using photolithography to mass-produce planar microrobots, it is conceivable that nanolithography techniques can be used to create planar nanorobots with biocompatibility, mobility, targeting capability, and size requirement for in vivo applications.

Current micro/nanorobots can be prepared using a variety of methods, such as biotemplating^24^, chemical conjugation^25,26^, photolithography^23^, glancing angle deposition (GLAD)^27^, or 2PP^10^. While each method offers reliable ways to fabricate various types of micro/nanorobots, they are not ideal for making planar nanorobots with consistency. To create achiral planar nanorobots, we proposed to use electron beam lithography (EBL) for its nanoscale resolution. There are currently no studies that used EBL to fabricate micro/nanorobots since the conventional EBL systems are limited to fabricating 2D structures and cannot realize mainstream 3D micro/nanorobot designs, such as helical robots. Thus, the 2D nature of the achiral planar design enables the possibility to use EBL to create nanorobots, hence providing a way to develop consistent magnetic nanorobots that can fulfill the size requirement for medical nanorobots in drug delivery.

To verify the potential to use these nanorobots for in vivo applications, they must be tested against the body’s first line of defense, the immune system. Macrophages are crucial immune cells that play a vital role in regenerative medicine, tumor-targeted therapy, and other biomedical fields; therefore, cytocompatibility with macrophages is an inevitable prerequisite for biomedical nanorobots. Most studies on medical micro/nanorobots ignored the role of the immune system; up until recently, several critical studies involving microrobots’ interactions with immune cells had propelled this issue to the forefront and established compatibility with the immune system as an essential design criterion for medical micro/nanorobots^28^. In this report, the achiral nanorobots were fabricated using EBL and are L-shaped with a minimum feature size of 400 nm. Control experiments were conducted to verify their swimming performance. Due to their small size, these nanorobots have the potential to be used for in vivo nanomedicine applications; to illustrate this potential, their cytocompatibility with cancer cells, normal cells, and immune cells and long-term stability in cell culture medium was studied.

## Results

### Design, fabrication, and characterization of nanorobots

**Figure 1:**
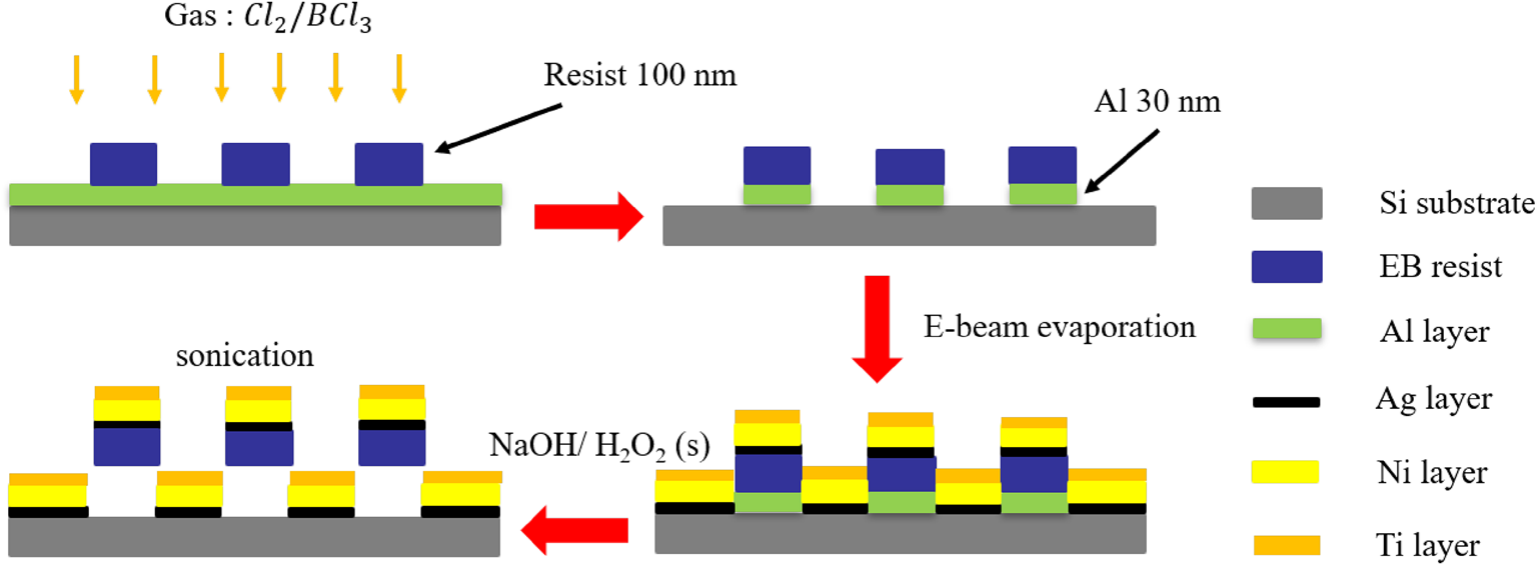
The preparation process of L-shaped nanorobots: (**a**) deposited Al layer through EBE, create resist patterns through EBL, and etch Al layer through ICP etching; (**b**) removed Al from regions not shielded by EB resist patterns; (**c**) deposited Ag, Ni, and Ti through EBE; (**d**) dissolved Al layer using NaOH and H_2_O_2_ solution to release the nanorobots.

The nanorobots were fabricated using electron beam exposure and electron beam evaporation methods. Using these methods, a large number of nanorobots can be fabricated on a silicon wafer, the minimum feature size of these nanorobots is about 400 nm. The fabrication process is shown in Fig. 1. The Ag layer acts as an adhesion layer between the Ni layer and the resist, the Ni layer endowed the nanorobots with magnetic properties, and the Ti layer made the nanorobots biocompatible. The end of the fabrication process yielded L-shaped nanorobots with the composition of resist/Ag/Ni/Ti anchored to the substrate by the Al sacrificial layer.

**Figure 2:**
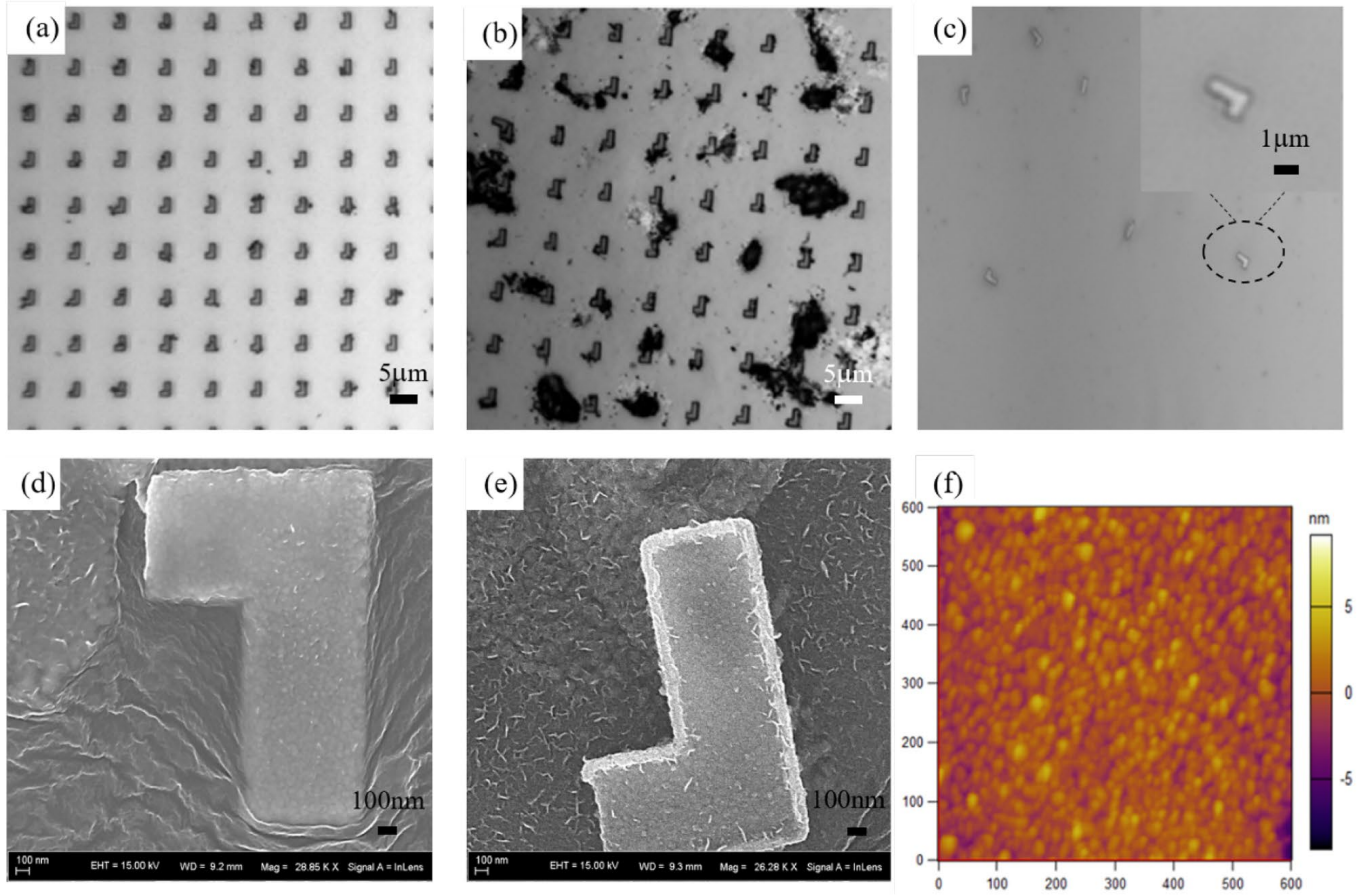
Characterization of the nanorobots. (**a**) microscope image of nanorobots before release. Nanorobots submerged in NaOH and H_2_O_2_ for (**b**) 10 min and (**c**) 20 min. SEM images of the (**d**) top and (**e**) bottom surfaces of the nanorobot. (**f**) AFM image of the granular Ti surface of a nanorobot; the granular surface can promote cell adhesion.

The release process is designed to allow for the release of nanorobots without releasing the excess Ag/Ni/Ti, thereby minimizing the amount of debris in the sample after releasing the nanorobots; this was possible since the excess sacrificial Al material was removed during the fabrication process. Figure 2a shows the microscopy image of nanorobots before release, Fig. 2d and e show SEM images of the nanorobot.

The Ti layer on the nanorobots was oxidized; it is a well-known phenomenon that metallic titanium is easily oxidized to form a thickness of about 10 nm titanium oxide due to natural oxidation^29^. As shown in Fig. 2f, the Ti layer on the L-shaped nanorobots after oxidization displays nanoparticle-like structures, which may promote cytocompatibility and cell adhesion^30^.

### Velocity measurement

**Figure 3:**
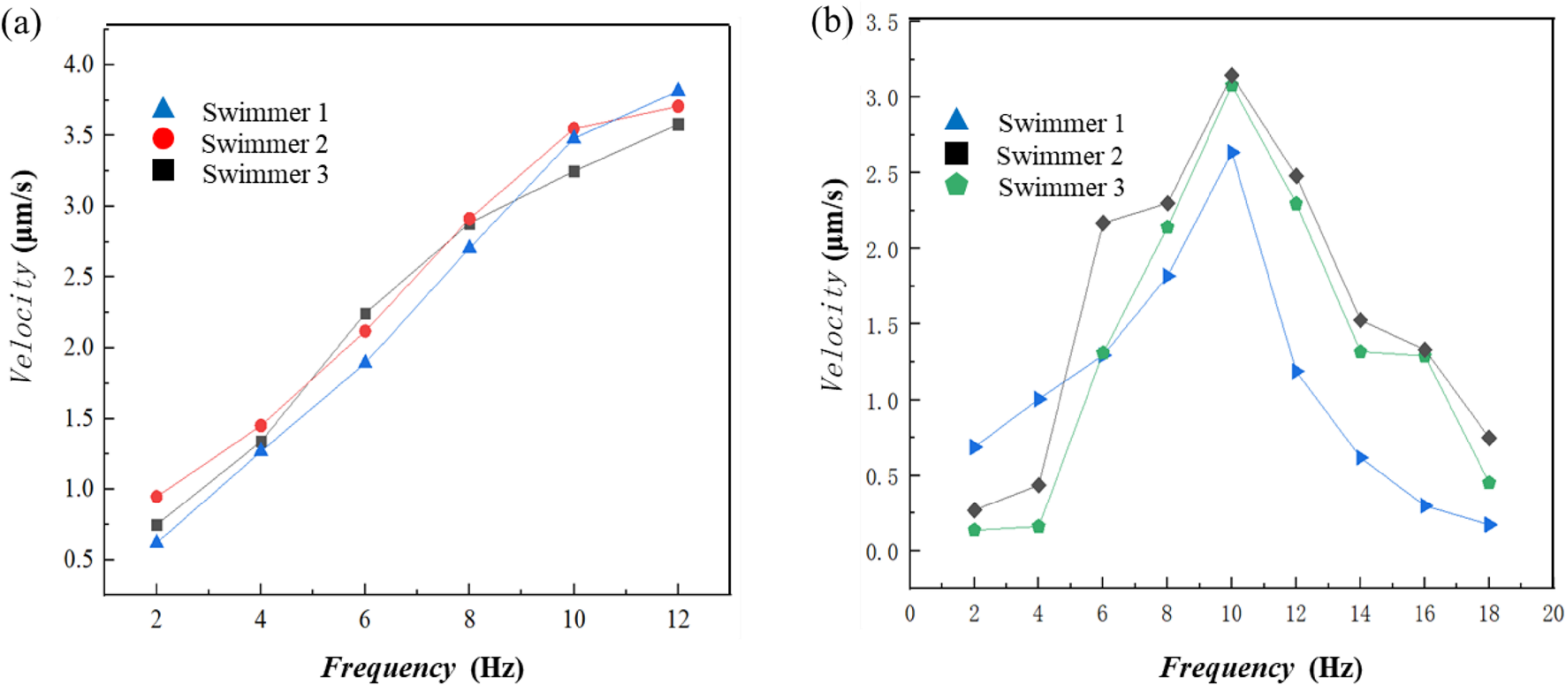
Velocity profiles of the nanorobots. (**a**) linear velocity profiles when the magnetic field frequency and strength increased proportionally. (**b**) nonlinear velocity profiles when the magnetic field strength remains constant at 2 mT while the frequency increases; step-out was observed at 10 Hz.

To test the swimming properties of the L-shaped nanorobots, a RMF generated by a three-dimensional Helmholtz coil was used to actuate their motion (see Fig. S1). The first test involved increasing the rotating frequency and strength of the magnetic field proportionally; this allows the nanorobots to maintain their same body-fixed rotation axis^20^. The average forward speed of the nanorobots increased with the magnetic field frequency, as shown in Fig. 3a and Movie S1. The second test involved keeping the field strength at 2 mT as the frequency increased; the nanorobots’ body-fixed rotation axis changed as the frequency increased, which led to a nonlinear relationship between speed and frequency^20^. Here we used MATLAB tracking algorithm to obtain the displacement over time of the nanorobots from several recorded videos (including Movies S1 and S2) and to calculate their average velocity ($v=\Delta d/\Delta t$). The nanorobots’ forward speed gradually increased until reaching a peak at 3.2 μm/s, then the nanorobots will step out, as shown in Fig. 3b and Movie S2. The sample size for each test was three, and the results are consistent across samples. These swimming behaviors are consistent with previously reported magnetic achiral microrobots^20^. The nanorobots, with a characteristic length of 1.4 μm and an average swimming speed of 3.2 μm/s at 10 Hz, have an average swimming efficiency of 0.23, which is on par with most existing micro/nanorobots. The swimming efficiency was characterized using dimensionless speed (${\nu }_{f}/Lf$)^23^.

### Cell cytotoxicity on normal cells and cancer cells

To demonstrate the potential to use the L-shaped nanorobots for in vivo applications, the cell toxicity of L-shaped nanorobots was tested on normal cells (Mouse fibroblasts L929) and cancer cells (HepG2 cells).

**Figure 4:**
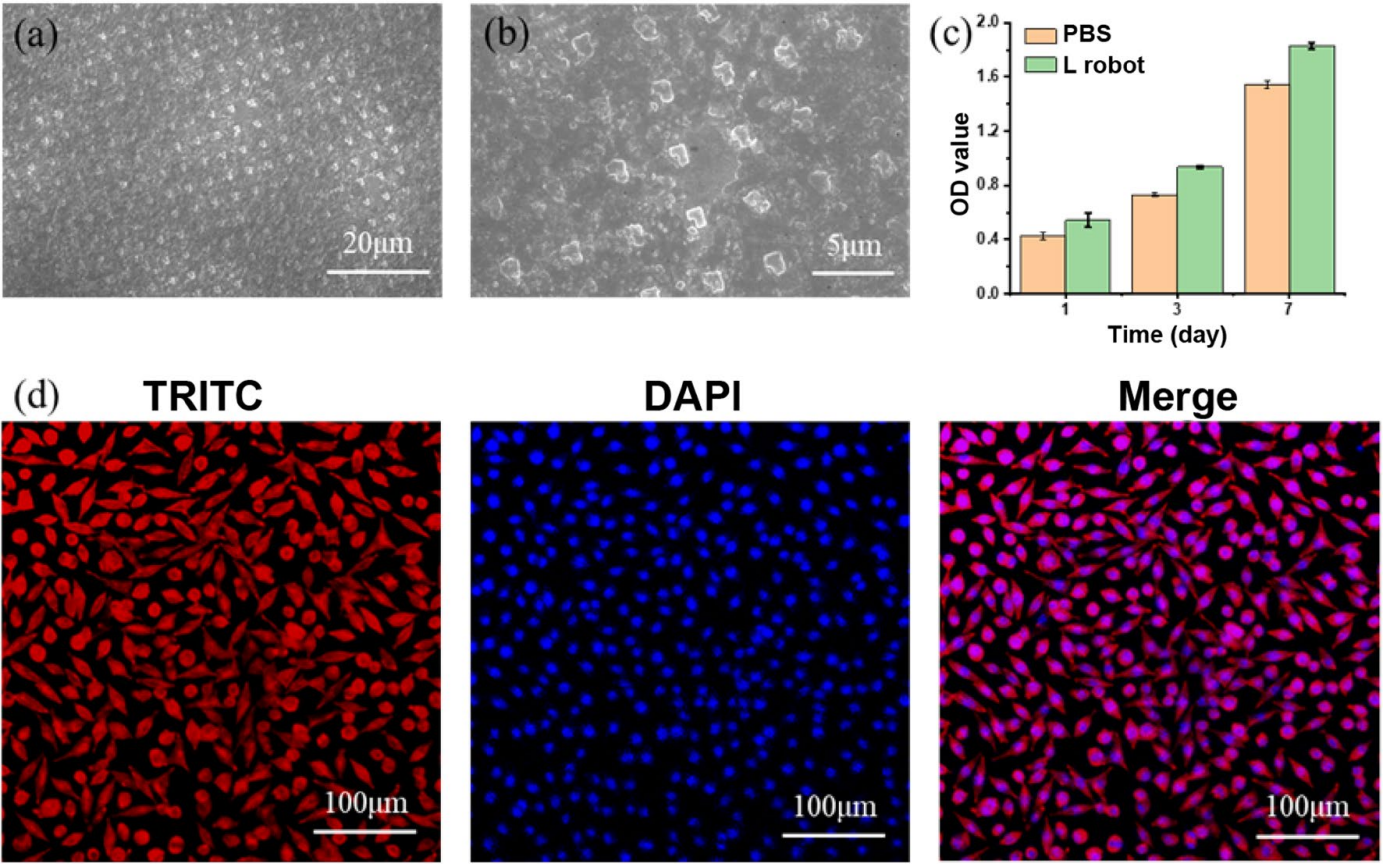
Cell cytotoxicity tests with mouse fibroblasts L929 cells. SEM image (**a**) and a zoom-in view (**b**) of L-shaped robot cultured with cells. (**c**) cell viability test using CCK-8 assay on days of 1 and 3. (**d**) Immunofluorescence staining, nuclei were stained with DAPI (blue), and F-actin was stained with TRITC-phalloidin (red).

The adhesion of L929 cells to the L-shaped nanorobots was examined on day 1 using SEM, as shown in Fig. 4a and b. CCK-8 assay was used to determine L929 cells cytotoxicity on days 1 and 3, as shown in Fig. 4c. The cells cultured on the L-shaped nanorobots showed good proliferation compared with the control groups. Filamentous actin (F-actin) stained by tetramethylrhodamine (TRITC)-phalloidin and nuclei stained by DAPI were imaged using confocal microscopy, as shown in Fig. 4d. These images indicate good interfacial adhesion between the nanorobots and cells.

**Figure 5:**
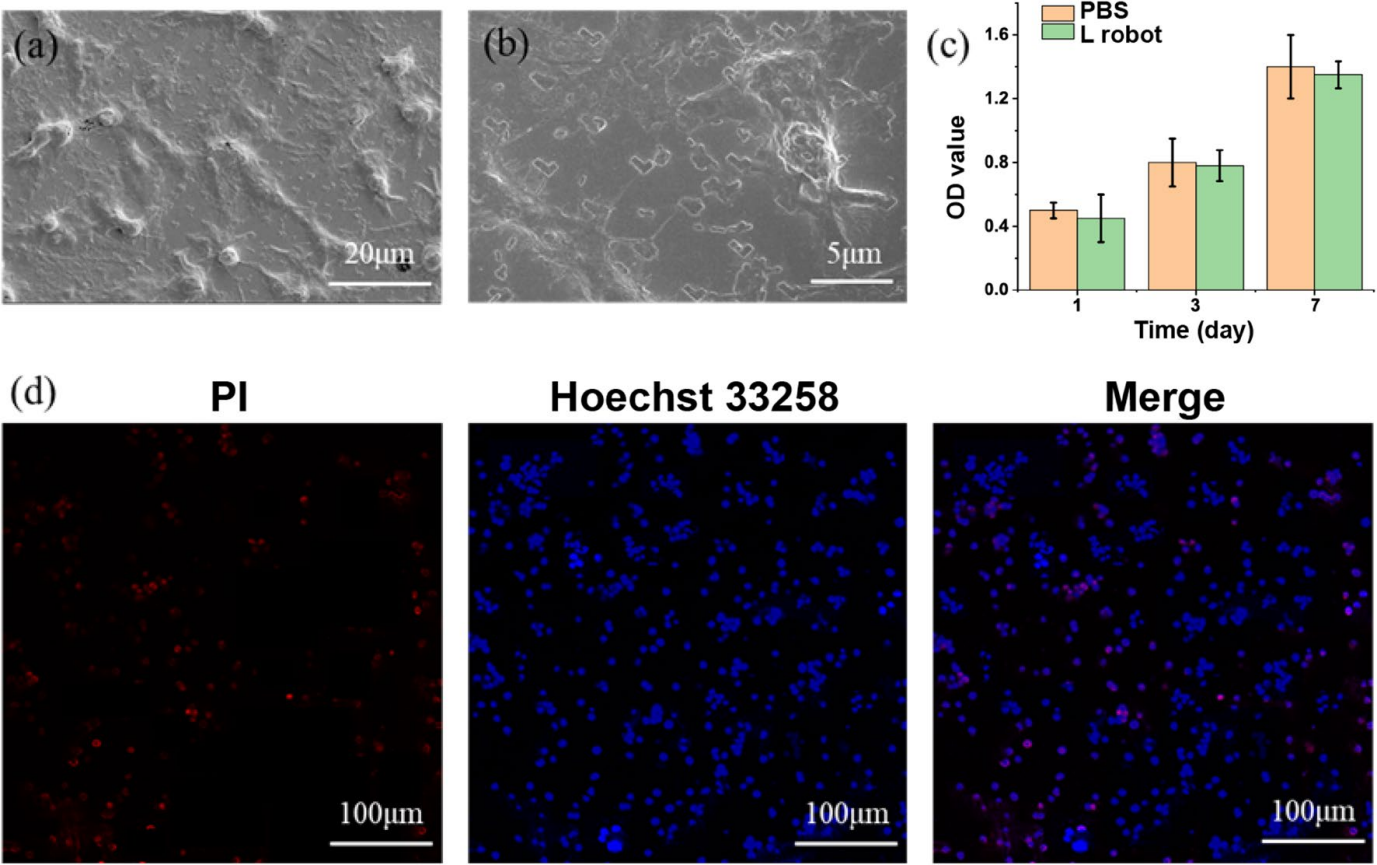
Cell cytotoxicity tests with HepG2 cancer cells. SEM image (**a**) and a zoom-in view (**b**) of L-shaped nanorobots cultured with HepG2 cancer cells. (**c**) Cell viability test using CCK-8 assay. (**d**) Live/dead by Hoechst 33258 (blue) /PI (red) staining.

For HepG2 cancer cells, no visible cell morphological abnormalities could be observed by SEM, as shown in Fig. 5a and b. Cell viability was also tested using CCK-8, and no significant cytotoxicity from the L-shaped nanorobots was observed in HepG2 cells compared to the control group, as shown in Fig. 5c. For live/dead staining (Hoechst 33258/PI), the necrotic cells, apoptotic cells, and normal cells were stained with high red /low blue fluorescent dye, high blue/low red fluorescent dye, and low blue/low red fluorescent dye, respectively. A tiny amount of red and high blue fluorescence spots were detected on tumor cells after a 1 and 3 days incubation with L-shaped nanorobots, as shown in Fig. 5d. These results suggested that the L-shaped nanorobots did not exhibit significant cytotoxicity on normal cells or cancer cells.

### Cell cytotoxicity on immune cells

**Figure 6:**
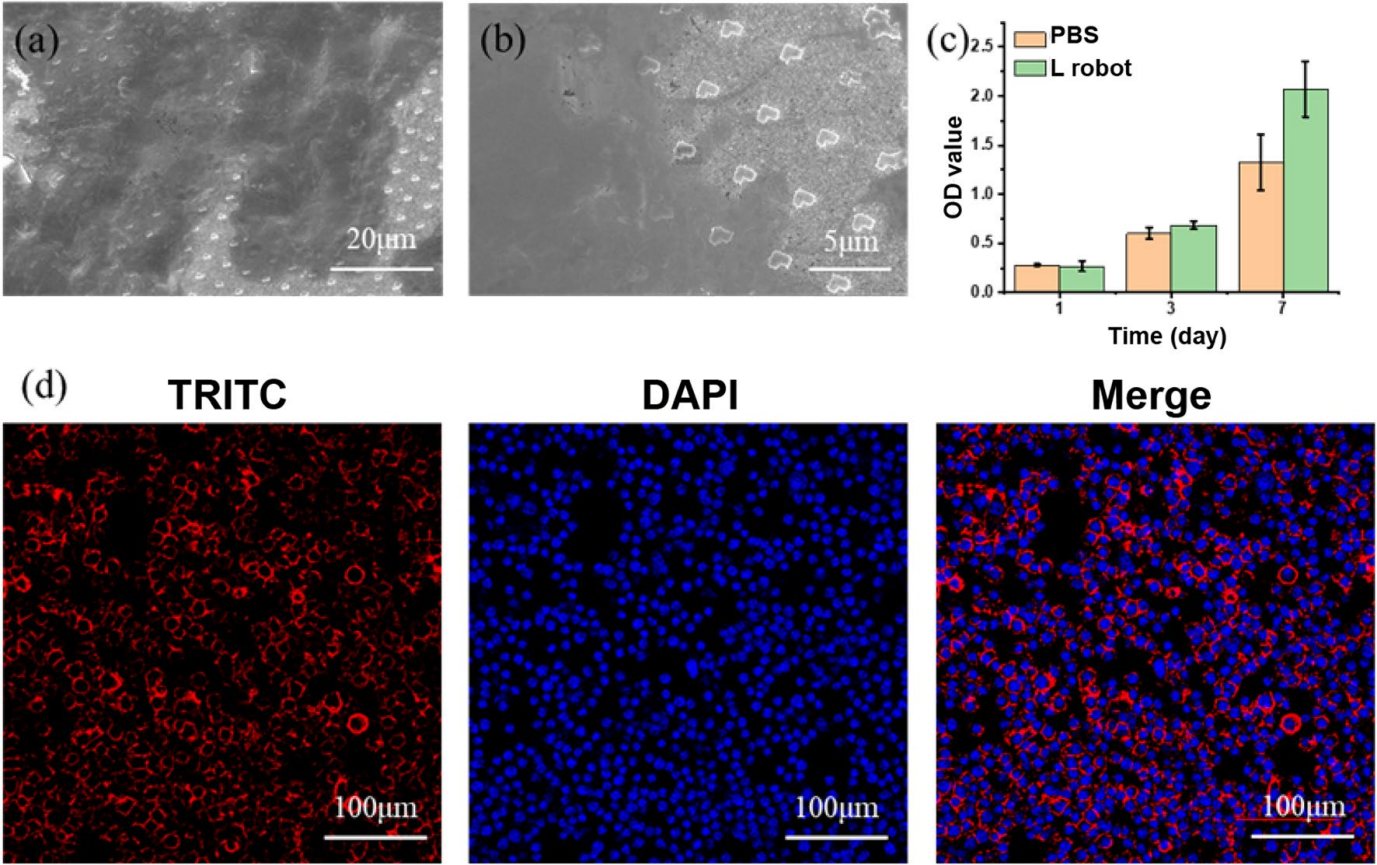
Cell cytotoxicitys with RAW264.7 immune cells. SEM image (**a**) and a zoom-in view (**b**) of L-shaped robot cultured with RAW264.7 immune cells. (**c**) Cell viability test using CCK-8 assay. (**d**) Immunofluorescence staining, nuclei were stained with DAPI (blue), and F-actin was stained with TRITC-phalloidin (red).

Macrophages cultured on L-shaped nanorobots were spread well and exhibited a relatively round morphology, as shown in the SEM and CLSM images in Fig. 6a and b. The CCK-8 results showed that the nanorobots had no inhibitory effect on the proliferation of the macrophages compared with the control group without nanorobots after 1 and 3 days, as shown in Fig. 6c. This indicates that the nanorobots can promote the proliferation of macrophages; this can synergize very well with regenerative medicine applications since more macrophage secretory factors and proteins need to be recruited in the early stage to promote regeneration and repair. Furthermore, the biocompatibility with macrophages indicates the potential for cancer treatment since specific macrophage phenotypes, such as the pro-inflammatory phenotype, are conducive to anti-tumor therapy and might play a positive role in the early diagnosis and treatment of tumors.

## Conclusion

In conclusion, we fabricated magnetic nanorobots using electron beam lithography and electron beam evaporation, verified their high propulsion efficiency under a RMF, and tested their biocompatibility. The planar design of the nanorobots enabled the possibility to scale the robots down to the nanoscale. In the speed characterization tests, the nanorobots achieved a swimming efficiency on par with most existing micro/nanorobots, which indicated that the planar L-shape design did not sacrifice locomotive capability. In in vitro experiments, the L-shape nanorobots did not exhibit significant cytotoxicity to HepG2 cells, indicating that the nanorobots did not affect cell proliferation. In addition, the biocompatibility of the nanorobot with macrophages and fibroblasts was proven, demonstrating that the nanorobots can promote cell proliferation. The nanorobots' small size, strong propulsive capability, and biocompatibility indicated their feasibility for targeted drug delivery and can play a positive role in applications such as early diagnosis and treatment of tumors.

## Experimental section

### Materials

AR-N 7520 e-beam resist and AR 300-46 development were bought from Allresist (Germany). Fetal bovine serum (FBS), high glucose Dulbecco's modified Eagle's medium (DMEM), RPMI-1640 medium, 0.25% Trypsin–EDTA, and penicillin–streptomycin were bought from Biological Industries (Kibbutz Beit-Haemek, Israel). Cell Counting Kit-8(CCK8) was purchased from MedChemExpress (USA). TRITC Phalloidin and DAPI were purchased from Soledad Bao Technology Co. (Beijing, China). Mouse fibroblasts L929, RAW 264.7 macrophages, HepG2 liver cancer cells were ordered from the Chinese Academy of Science cell bank.

### Fabrication of nanorobots

The fabrication process of the nanorobots is shown in Fig. 1. First, a 30 nm sacrificial aluminum (Al) layer was evaporated on a silicon wafer with the deposition speed of 6 Å/s . Next, a 100 nm layer of the electron beam (EB) resist (AR-N 7520.07) was deposited through spin coating with a coating speed of 3000 rpm followed by baking at 95 °C for 3 min. Then, the sample was exposed to an electron beam exposure unit with an exposure measurement of 750 u/cm^2^ to create L-shape patterns, as shown in Fig. 1a. After that, the sample was developed through inductively coupled plasma (ICP) etching for 20 s under 300 W to remove the excess Al material not shielded by the EB resist, as shown in Fig. 1b. Finally, the sample was coated with 20 nm silver (Ag), 70 nm nickel (Ni), and 10 nm titanium (Ti) layers through electron beam evaporation (EBE), as shown in Fig. 1c; the coating speed for these three materials were 4 Å/s, 6 Å/s, and 6 Å/s respectively.

The sample was placed in a mixed solution of sodium hydroxide (NaOH) and hydrogen peroxide (H_2_O_2_) at 65 °C for 5 min to dissolve the Al sacrificial layer, as shown in Fig. 1d. The H_2_O_2_ will decompose under heating to produce bubbles to push the nanorobots off the substrate, allowing the nanorobots to be dispersed in the solution after release. The nanorobots were observed before submersion in NaOH and H_2_O_2_, after 10 min of submersion, and after 20 min of submersion, as shown in Fig. 2a–c respectively. It was observed that the nanorobots were fully released and dispersed in the solution without aggregation after 20 min of submersion, as shown in Fig. 2c.

### Cell culture

Cell cultures were maintained at 37 °C, 5% CO_2_. Mouse fibroblasts L929 and RAW 264.7 macrophages were cultured in DMEM supplemented with 10% fetal bovine serum and 1% penicillin–streptomycin. HepG2 liver cancer cells were cultured in RPMI-1640 medium, supplemented with 10% fetal bovine serum and 1% penicillin–streptomycin. Media was changed every 48 h, and cells were passaged with 0.25% Trypsin–EDTA with 1 min incubation at 37 °C.

### Cytotoxicity evaluation

Cell cytotoxicity was determined using a CCK-8 assay. Samples were sterilized by autoclaving at 121 °C, 20 min. Cells suspension with a density of 2 × 10^4^ cells per ml were seeded on the samples. The cell's cytotoxicity was measured at different time points according to the following steps. The cells incubated with samples were cleaned with PBS, and then a fresh complete DMEM medium containing 10 v% CCK-8 was added. Two hours later, solutions of 100 µL for each sample were transferred to a 96-well culture plate, and the absorbance was measured at 450 nm by an enzyme-labeling instrument (Tecan).

### Cell morphology by SEM and immunofluorescence

Cells were exposed to samples in a 48-well cell culture plate. After 24 h, cells were washed three times with phosphate-buffered saline (PBS) and fixed with 4% paraformaldehyde for 15 min at room temperature and followed by washing with PBS. For SEM, cells were dehydrated through graded ethanol (30 to 100% v/v) and air-dried.

For immunofluorescence staining, cells were then permeabilized with 0.1% Triton X-100 for 15 min at room temperature, washed three times (5 min each) with PBS. The actin filaments were labeled with tetramethylrhodamine (TRITC)-labeled phalloidin for 40 min, and cell nuclei were stained with DAPI for 5 min. The stained cells were washed and sealed with an anti-fluorescence quenching agent. Imaging was performed on Nikon A1R Confocal Microscope.

### Live–dead cell staining

The live–dead cell staining was performed using Hoechst 33258 and propidium iodide assays. Briefly, cells were cultured on the specimens with a density of 1 × 10^6^ cells per well. Hoechst 33258 and propidium iodide and calcium-AM were diluted to final concentrations of 10 μg/mL in PBS, respectively. After one day, 100 μL of the mixed solution was added to each specimen, and the cells were stained at 37 °C for 10 min.

## Supplementary Information

Supplementary Figure S1.

Supplementary Movie S1.

Supplementary Movie S2.

## Data Availability

All data generated or analysed during this study are included in this published article and its supplementary information files.
