# Supplementary material for: Macrophage-compatible magnetic achiral nanorobots fabricated by electron beam lithography
Source: Sci Rep. 2022 Jul 29;12:13080. doi: 10.1038/s41598-022-17053-x (PMC9338296; doi:10.1038/s41598-022-17053-x)
Supplement: Supplementary file 1 — Supplementary Figure S1. [file 41598_2022_17053_MOESM1_ESM.doc]

## Macrophage-Compatible Magnetic Achiral Nanorobots Fabricated by Electron Beam Lithography

*Teng Jiang^#,a^, Xiaoxia Song^#,a^, Xueliang Mu^d^, U Kei Cheang^*,a,b,c^*

^a^Department of Mechanical and Energy Engineering, Southern University of Science and Technology, Shenzhen 518055, China

^b^Shenzhen Key Laboratory of Biomimetic Robotics and Intelligent Systems, Southern University of Science and Technology, Shenzhen, China

^c^Guangdong Provincial Key Laboratory of Human-Augmentation and Rehabilitation Robotics in Universities, Southern University of Science and Technology, Shenzhen, China

^d^Department of Mechanical and Manufacturing Engineering, University of Calgary, Calgary, Alberta, Canada

#These authors contributed equally: Teng Jiang and Xiaoxia Song

*Corresponding authors
**E-mail:** cheanguk@sustech.edu.cn

In this study, a rotating magnetic field generated by a three-dimensional Helmholtz coil is used to control the motion of the L-shaped robot. As shown in Figure S1, a chamber is made of the glass sheet and PDMS, and the chamber with a nanorobot is placed in the middle of the coil. Then we open the power supply to start the magnetic field to control nanorobots.


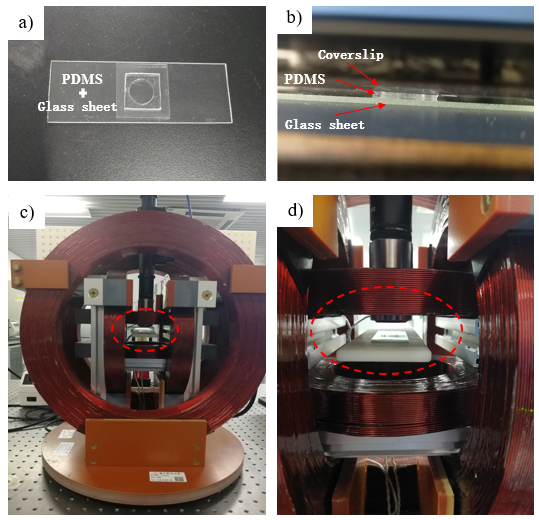


**Figure S1.** (a) The top view of the cavity (glass sheet and PDMS) containing the nanorobot, (b) The side view of the cavity containing the nanorobot, (c) The front view of the position of the cavity in the coil, and (d) The position of the cavity in the coil.
